# Efficacy of “Family Connections”, a program for relatives of people with borderline personality disorder, in the Spanish population: study protocol for a randomized controlled trial

**DOI:** 10.1186/s12888-020-02708-8

**Published:** 2020-06-15

**Authors:** Isabel Fernández-Felipe, Verónica Guillén, Helio Marco, Amanda Díaz-García, Cristina Botella, Mercedes Jorquera, Rosa Baños, Azucena García-Palacios

**Affiliations:** 1 Universitat Jaume I, Castellón, Spain; 2 Universidad de Valencia, Valencia, Spain; 3 ITA-PREVI (Personality Disorders Centre), Castellón, Valencia and Alicante Spain

**Keywords:** Borderline personality disorder, Family connections, Relatives, DBT, Intervention, Caregivers, Burden

## Abstract

**Background:**

Patients with borderline personality disorder (BPD) experience significant affect regulation difficulties that cause serious consequences in their work, emotional, and social environments. This dysfunctional pattern also produces great suffering and a heavy burden on their relatives. Fortunately, some studies show that treatment of relatives of people with BPD begins to be important in the patients’ recovery and in improving family dynamics. One of the treatments that has obtained the most empirical support is Family connections (FC). This 12-session program is an adaptation of different Dialectical Behavior Therapy strategies. To test the efficacy of FC, five uncontrolled clinical trials were conducted, with pre-post treatment and follow-up assessments. The results of these studies and subsequent replications showed an improvement in family attitudes and caregiver burnout. Our research team adapted FC for delivery in the Spanish population. We intend to test the efficacy of this program versus a treatment as usual condition. Moreover, we aim to test the efficacy of this program and study its effectiveness (in terms of participants’ acceptance). This paper presents the study protocol.

**Methods:**

The study is a randomized controlled trial. The participants will be recruited in a Personality Disorders Unit and randomly assigned to one of two treatment conditions: *Family Connections group (FC)* or Treatment As Usual (TAU). Primary outcome measures will be the BAS and FAD-GFS. Secondary outcomes will include DASS-21, FES, GS, and QLI. Participants’ treatment acceptance and degree of satisfaction will also be measured. Participants will be assessed at pre-, post-treatment, and 6-month follow-up. Intention to treat and per protocol analyses will be performed.

**Discussion:**

This is the first study on FC for relatives of people with borderline personality disorder (BPD) compared to an active condition (TAU), and this is the first time relatives’ and patients’ data will be analyzed. In addition, it is the first study to test the efficacy of the program in Spain. This intervention could contribute to improving the efficiency and effectiveness of current treatment programs for relatives of people with BPD, help to decrease burden, and improve the family connection.

**Trial registration:**

ClinicalTrials.gov ID: NCT04160871. Registered November 15th 2019.

## Background

Borderline personality disorder (BPD) is one of the most challenging and complex mental disorders. BPD is related to high suicide and self-harm rates. Persistent suicidal behavior is described in 69–80% of people with BPD [1]. A longitudinal study across 24 years comparing BPD and other personality disorders found that a total of 5.9% of BPD sufferers died by suicide and 14% by other causes, compared to 1.4 and 5.5% in a sample of people with other personality disorders [2]. BPD also involves high rates of hospital admissions and health service use. BPD is associated with a high economic burden due to the long-term use of health services [3–6], including interventions in emergency settings and the need for the services of several different professionals [6–8]. Furthermore, BPD is an important public mental health problem that produces great suffering for patients and their relatives [9]. For this reason, there is a need to provide specialized care.

The symptoms of BPD and their consequences lead to high levels of discomfort and burden for their relatives [10–14]. Additionally, there is evidence that maladaptive family communication patterns play a role in the etiology and maintenance of BPD [14, 15].

Family members of people with BPD are more likely to have psychological problems [16, 17], and they describe feelings of confusion, lack of awareness, and incompetence [14, 18, 19]. Studies with relatives of people with BPD showed that the levels of burden and depression can increase due to lack of clear knowledge about the diagnosis and the evolution of the disorder [19, 20]. Moreover, when family members participate in treatment, patient relapse decreases, recovery is easier, and wellbeing in the family improves [20, 21].

Currently, there are interventions for family members with empirical support. All these programs are offered in group format, but they differ in the type of orientation and contents. So far, two of these studies present only psychoeducational contents; one is based on mentalization [22], and the other combines cognitive analytical therapy with general psychiatric care [23]. Regarding the programs that offer skills training, almost all are DBT-based programs or DBT adaptations. These DBT skills training studies have different structures and numbers of sessions. They use either adaptations of DBT in 10–12 sessions where parents receive training in DBT mini-skills [17, 24–27] or group therapy where skills are taught for 6 months [28].

Family Connections (FC) is the most empirically supported program [25] for relatives of patients with BPD. The program can be carried out by clinicians or trained relatives. To date, five uncontrolled clinical trials have been performed with pre- and post-treatment and follow-up assessments [17, 24, 25, 29, 30]. In all the replications, the results of the FC program were consistent, showing significant decreases in burden, grief, anxiety, and depression, and significant increases in the participants’ subjective experience of mastery, empowerment, well-being variables, and family functioning. Furthermore, these variations were maintained or improved at 3- or 6-month follow up. The good results for family functioning could be due to the fact that FC validates patients’ skillful behaviors, decreases their psychological symptoms, improves interpersonal relationships between family members and patients, increases understanding of the problem, reduces perceived stigma, and enhances family empowerment [30].

FC is a program for relatives of people with BPD that was developed within the National Education Alliance for Borderline Personality Disorder [17, 25]. This program links three important needs for relatives: first, education about the disorder and family functioning; second, individual and family skills to manage negative reactions in the family and improve well-being in the relationship; and, finally, social support from other relatives participating in the same group who have had very similar experiences [17].

A pilot study by Hoffman et al. [25], with pre-, post-, and 6-month follow-up of one group, suggests that this program promotes significant reductions in grief and burden and a significant increase in mastery. A replication and extension study of FC by Hoffman et al. [17], with a pre-, post-, and 3-month follow-up of one group, shows improvements in well-being variables and depression. Another descriptive mixed study (qualitative and quantitative data) with two groups (family members with and without clinically relevant symptoms) showed that the subgroup with clinically relevant symptoms had a significant decrease in depression and anxiety symptoms at follow-up, and women showed a decrease in both anxiety and depression symptoms before and after the intervention [29]. Flynn et al. [24] found similar results in a non-randomized controlled study (pre-, post-, 3-, 12-, or 19-month follow-up) that compared FC with a psychoeducation group. Finally, in a non-randomized comparison study with pre-, post-, and 6-month follow-up assessments, participants who received FC reported fewer mental health difficulties, a lower perceived burden of caring, and higher overall family functioning [30].

Therefore, considerable progress has been made in this line of work, which had not previously been considered. However, it would be desirable to advance in this direction by comparing FC to active treatments in larger samples and, if possible, examine the impact of the treatment on both family members and patients. Another important issue is the dissemination of FC to other cultural contexts. This study will provide the first efficacy data on the comparison of FC with an active treatment condition in a randomized controlled trial. Another contribution of this study is the measurement of the evolution of the family climate in relation to the improvements of both relatives and patients. Finally, this is the first study on FC carried out in a Spanish-speaking population.

This study has several objectives. First, we aim to test the efficacy of FC for relatives of patients with BDP in an RCT with a sample of participants from specialized care in Spain, compared to Treatment as Usual (TAU), that is, an active treatment condition. Second, we will study the feasibility and acceptance of this intervention protocol in family members of patients with BPD. Third, we intend to study whether changes in family members’ disease burden and clinical symptoms are related to the improvements observed in patients with BPD. Fourth, we aim to study whether the changes that may occur in relatives with regard to disease burden and clinical symptoms are related to the family climate. Finally, we will study the perceptions and opinions of families and patients about both intervention protocols.

We hypothesize that: a) both interventions will result in significant reductions in distress and burden and improvements in overall family functioning at post-treatment, and these results will be maintained at the 6-month follow-up; b) the FC program will significantly outperform the TAU intervention on measures of subjective burden, validation skills, family functioning, and quality of life; c) both protocols will be well accepted, but FC will be rated significantly higher by the participants; d) the improvement that may occur in the family members with regard to disease burden and clinical symptoms will have a positive influence on the family climate; e) in an exploratory way, given the lack of specific data in the literature, we hypothesize that the changes observed in the relatives will be related to the clinical evolution of the patients. In this article, we present the study protocol.

## Methods

### Study design

**Fig. 1:**
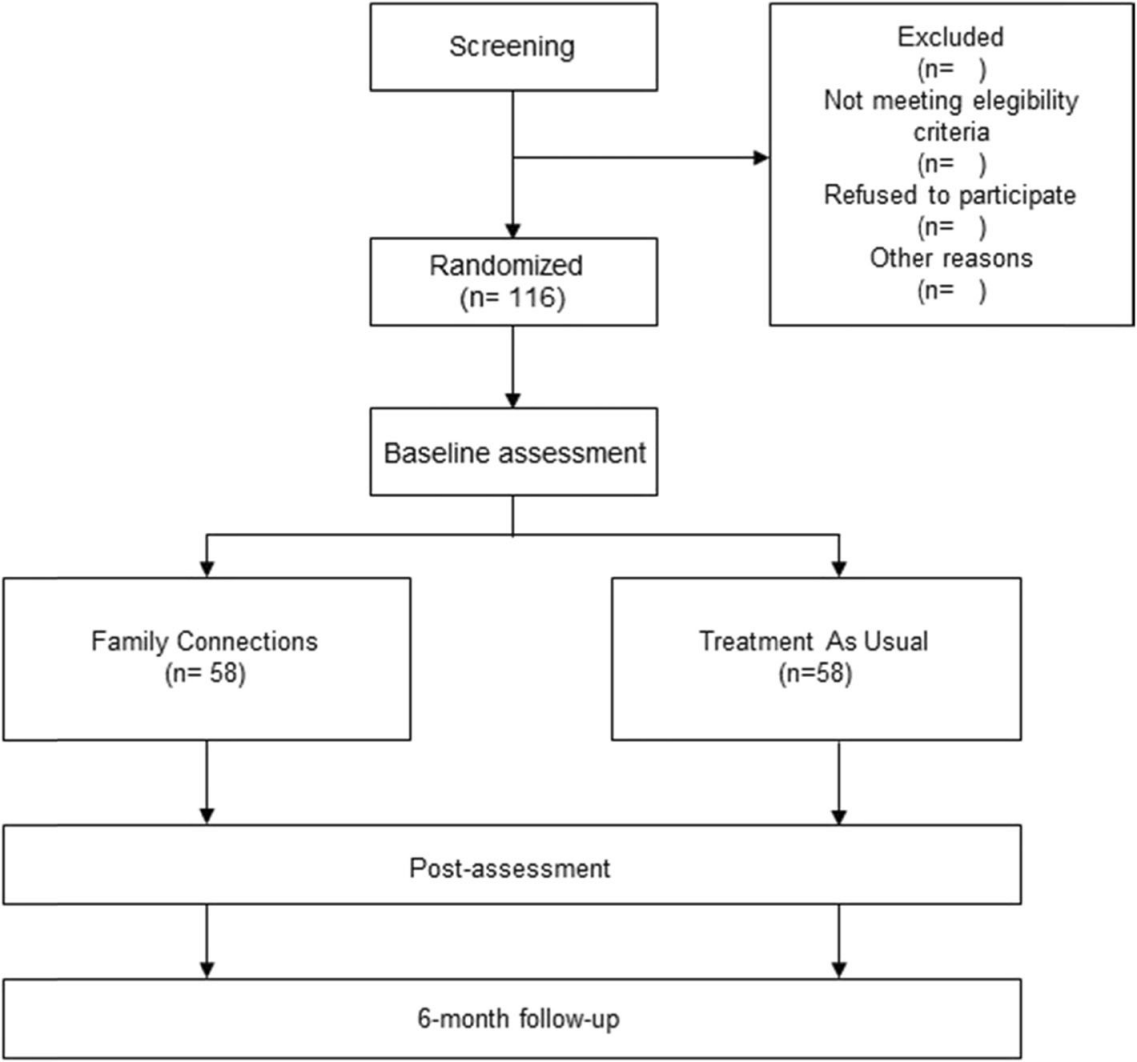
Flowchart of the study

We will conduct a two-armed randomized controlled trial (RCT). Participants will be randomly assigned to one of two conditions: Family Connections (FC) or Treatment As Usual (TAU). Block randomization will be carried out among the three clinical centers, considering that if a patient has more than one family member who attends the group, they will be randomized together to be included in the same condition. Measures will be taken before starting the intervention, after the intervention, and at the 6-month follow-up to determine whether improvements after the intervention are maintained in the long term. The study flowchart appears in Fig. 1. We will follow the CONSORT statement (Consolidated Standards of Reporting Trials, http://www.consort-statement.org) [31, 32] and the SPIRIT guidelines (Standard Protocol Items: Recommendations for Interventional Trials) [33, 34].

### Sample size

To determine the sample size, the effect sizes found in the literature on the subject have been considered. The controlled study by Grenyer et al. [35], which tested a group psychoeducation protocol for caregivers of people with BPD, reported medium to large effect sizes (dyadic adjustment, *d* = .78; family empowerment, *d* = 1.4). In addition, on measures of burden, Grenyer [35] reported significant improvements between post assessment and the 12-month follow-up, with medium effect sizes (Burden Assessment Scale, *d* = .45). These effects are consistent with the literature on psychological treatments for other psychiatric disorders, such as the meta-analysis of psychological interventions for caregivers of people with bipolar disorders (Burden, *g* = −.80) [36].

Taking these data into account, in the present study, adopting a conservative approach, an effect size of 0.60 is expected because our design includes two experimental conditions. Considering an alpha of 0.05 and a statistical power of 0.80 on a 2-tailed *t* test, the total sample size needed to reach an effect size of 0.60 for burden is 90 participants (45 participants per experimental condition). To control the maximum possible loss of participants during treatment, based on the literature about programs for family members of patients with BPD, a 29% dropout rate is expected [20, 23–25, 27]. Thus, the required sample size should be a total of 116 participants (58 participants per group). These calculations were made with the software program G*Power 3.1 [37].

### Study population, recruitment, and eligibility criteria

The sample will consist of relatives of people with BPD. Recruitment will be carried out among relatives of patients treated at clinical centers specializing in the treatment of BPD in the Valencian region. Inclusion criteria will include the following: a) being 18 years old or more; b) having a family member diagnosed with BPD; c) ability to understand and read Spanish; and d) providing written informed consent. Participants will be recruited by clinicians working in these clinical centers in three Spanish cities (Castellón, Valencia, and Alicante), until the required sample is complete. Clinicians will offer patients’ families the opportunity to participate in the study and, after explaining it, obtain their informed consent. All the psychologists who participate in this research will have at least a master’s degree in Clinical Psychology and specialized FC training.

A psychologist will contact the participant to determine his/her inclusion in the study. At that time, the researcher will collect the baseline data and determine whether the inclusion criteria are met (see Table 1). Then the psychologist will contact a person outside the research group who will perform the individual randomization and inform the assessor of a code that corresponds to the type of treatment. This psychologist will be unaware of the characteristics of the study.

**Table 1:** Study measures and evaluation times

Participant	Measure	Aim	Evaluation time
Caregiver	S-D Interview	Diagnosis	BL
	BAS	Severity of burden symptoms	BL, Post-T and FU
	FAD-GFS	Familiar Global Functioning	BL, Post-T and FU
	DASS-21	Depression, anxiety and stress symptoms	BL, Post-T and FU
	FES	Family empowerment	BL, Post-T and FU
	QLI-Sp	Quality of life	BL, Post-T and FU
	OTSM	Treatment opinion and acceptance	PM
Patient	FAD-GFS	Familiar Global Functioning	BL, Post-T and FU
	DASS-21	Depression, anxiety and stress symptoms	BL, Post-T and FU
	DERS	Difficulties in emotional regulation	BL, Post-T and FU
	LEAP	Emotional availability of parents	BL, Post-T and FU
	VIRS	Validating and invalidating responses	BL, Post-T and FU

*BL* Baseline; *Post-T* Post-treatment; *FU* 6-month follow up; *OTSM* Opinion of Treatment Scale by Modules; *S-D interview* Socio-Demographic Interview; *BAS* Burden Assessment Scale; *FAD-GFS* Family Assessment Device – Global Functioning Scale; *DASS-21* Depression, Anxiety and Stress Scale; *DERS* Difficulties in Emotion Regulation; *FES* Family Empowerment Scale; *QLI-SP* Quality Life Inventory-Spanish version; *GS* Grief Scale; *LEAP* Lum Emotional Availability of Parents; *VIRS* Validating and Invalidating Responses Scale

The psychologist will ensure that the participant has understood the characteristics of the study, and he/she will answer any questions the participant has. Participants will agree (or not) to participate before knowing which intervention condition they will be assigned to. The participants will also be informed that they can leave the study whenever they wish, and that in no case will there be any negative consequences for their family member who is receiving treatment at the center.

The psychologists who will participate in this study have extensive experience in implementing the DBT program for patients and will receive training in the FC program.

### Ethics

The study will follow the Declaration of Helsinki Guidelines and existing guidelines in Spain and the European Union for the protection of participants in clinical trials. The Ethics Committee of the University of Valencia (Valencia, Spain) has approved this study. The trial was registered at clinicalstrial.gov as NCT04160871, registered the 15th of November of 2019.

Sample recruitment will be carried out by qualified clinicians. Researchers will explain the study to the participants, and they will sign the consent form as volunteer participants with the possibility of dropping out at any time. If our hypotheses are confirmed, the FC condition will be offered to participants assigned to the TAU condition after the 6-month follow-up. Special difficulties are not expected, based on the literature. If a participant drops out of the trial due to unwanted events, s/he will have the opportunity to participate the next time the treatment groups for family members are offered.

To protect information, personal data (e.g. age, sex, address, mail, phone) will be collected by the researchers participating in this study, and data will be replaced by codes. Personal data will be strictly separated from other data, and it will only be available to researchers responsible for the study, always considering and protecting the right to privacy of the participants.

### Interventions

We translated the FC protocol for relatives of people with BPD into Spanish. It is one of the first programs designed to be applied directly to relatives of patients with BPD. The program is an adaptation of different Dialectical Behavior Therapy strategies, one of the most researched and empirically supported treatments for BPD people [24, 38]. It is composed of six modules divided into 12 sessions lasting approximately 2 h each. The intervention protocol is structured in a caregiver handbook [25]. In the following section, the modules in each treatment program are briefly described.

The FC protocol includes components aimed primarily at reducing distress and burden and improving overall family functioning: relationship mindfulness skills, family environment skills, validation skills, and problem management skills. Furthermore, the program includes Psychoeducation about borderline personality disorder.

#### Family connections (FC)

This intervention program consists of six modules with two sessions each, designed to improve family attitudes and reduce family exhaustion. Each module has specific objectives and practical exercises, as well as videos with examples of people suffering from BPD and their relatives:

1. *Introduction*. The objective of this module is to provide information about the aims of the program, weekly format and guidelines, statement of rights, and criteria and symptoms of BPD. The central role of emotion regulation is also presented.
2. *Family Education*. The purpose of this module is to present treatment programs for BPD and comorbid disorders, biosocial factors related to the etiology of the disorder, the difficulties BPD provokes in the family members, and the need for help. It also shows the transactional model of the development of BPD and related disorders.
3. *Relationship Mindfulness Skills*. This module aims to define a validating family environment, being mindful of the relationship, emotion regulation skills, and states of mind.
4. *Family Environment Skills*. The aim is to understand the relationship between the individual and the family’s welfare, the importance of maladaptive ways of thinking related to blame, and the concept of radical acceptance.
5. *Validation Skills*. The objective of this module is to understand what validation means and learn validation and self-validation skills. Moreover, in this module, the relatives learn how to set clear limits and achieve self-respect.
6. *Problem Management Skills*. This module focuses on interpersonal efficacy, defining problems and solutions, and problem management skills.

### Adaptation to Spanish

The FC program has been translated into Spanish by the Puerto Rican research group directed by Dr. Domingo Marqués, and adapted to the Spanish spoken in Spain by our research team. This translation was performed by clinical experts who were familiar with both DBT [39, 40] and the FC program. The translation included the FC program manual, as well as the videos that accompany the program (they were subtitled in Spanish) and the brochures, leaflets, and handouts.

#### Treatment as usual (TAU)

Treatment as usual is the program routinely offered to BPD patients’ relatives in the clinical centers participating in this trial. The intervention includes 12 therapeutic sessions in six modules. Each module has specific objectives and practical exercises.

1. *Introduction*. This module consists of an overview of the treatment and the aims of the group. Furthermore, it focuses on the definition of personality disorders, BPD and its clinical characteristics, the role of emotion regulation, and comorbid disorders.
2. *Family Education*. The aim of this module is to explain the diagnostic criteria for BPD, associated problems (alcohol and substance use and eating disorders), the DBT model, and the main goals of the treatment.
3. Validation Skills. The purpose of this module is to explain what validating and invalidating environments are, the consequences of an invalidating environment, and how to use validating skills.
4. *Crisis Management Skills*. This module aims to prevent crises by explaining how to manage anger and learning how to act in the presence of self-injuring and suicidal behaviors. Moreover, acceptance skills are shown in this module.
5. *Problem Management Skills*. This module helps the relative to know how to deal with problems and set clear limits, handle conflict in everyday situations, confront unacceptable behavior, and manage emotionally charged conversations.
6. *Relapse Prevention*. It aims to strengthen the strategies learned throughout the program, schedule future practice, and teach the participants how to identify and cope with future high-risk situations.

### Measures

Table 1 presents a summary of the measures.

### Caregiver measures (participants)

#### Sociodemographic interview

Demographic variables questionnaire: age, family constellation, sex, educational level, income, marital status, number / age of children, and psychiatric history.

### Primary outcomes

#### Burden assessment scale (BAS) [41]

It consists of 19 items that assess the caregivers’ objective and subjective burden in the past 6 months. Items are rated on a 4-point Likert scale ranging from 1 to 4, and higher values indicate a heavier burden. Internal reliability of the scale ranges from .89 to .91, and it shows adequate validity [42]. This scale is not validated in Spanish and it will be an objective of this work.

#### Family assessment device – global functioning scale (FAD-GFS) [43]

It is a self-report questionnaire consisting of 60 items related to family functioning. It is composed of seven subscales: Problem-Solving, Communication, Roles, Affective Responsiveness, Affective Involvement, Behavior Control, and General Functioning. Cronbach’s alphas range from .72 to .83 for the subscales, and.92 for general functioning, and test-retest reliabilities for the FAD scales were adequate [44]. This scale is not validated in Spanish and it will be an objective of this work.

### Secondary outcomes

#### Depression, anxiety and stress scale (DASS-21) [45]

This scale has 42 items about negative emotional symptoms. They proposed a short version with 21 items. The DASS-21 showed good factor structures. Regarding the internal consistency, Cronbach’s alphas were excellent for the DASS-21 subscales: Depression (α = .94), Anxiety (α = .87), and Stress (α = .91) [46]. We used the Spanish version validated by Daza, Novy, Stanley and Averill [47].

#### Family empowerment scale (FES) [48]

It consists of 34 items divided into three subscales: family, service system, and involvement in community, which refer to three forms of empowerment: attitudes, knowledge, and behaviors. Items are rated on a scale from 1 to 5, and higher scores indicate a greater sense of empowerment. The psychometric properties are the following: regarding the internal consistency of the FES subscales, the coefficients ranged from .87 to .88, and validity and reliability were adequate. This scale is not validated in Spanish and it will be an objective of this work.

#### Quality of life index-Spanish version (QLI-Sp) [49]

This index consists of 10 items that assess perceived quality of life, including physical and emotional well-being, self-care and independent functioning, occupational and interpersonal functioning, social-emotional and community support, personal and spiritual fulfillment, and a global perception of quality of life. Higher scores indicate higher quality of life. This instrument has good psychometric properties, with a Cronbach’s alpha of .89 and high test–retest reliability (*r* = 0.87).

#### Opinion of treatment scale by modules (OTSM)

The Opinion of Treatment Scale by Modules is an instrument developed by our research team and adapted from Borkovec and Nau [50]. It is designed to assess the participants’ opinion and acceptance of the program. Furthermore, it evaluates the level of change obtained with regard to the therapeutic modules. Questions involve how logical the treatment seemed, degree of satisfaction, if they would recommend the program, if they think this program would be useful to treat their problems or others, and expectations about the program. It evaluates the six treatment modules in the two conditions. There are two subscales: one evaluates the learning of the skills taught in the module and is rated from 0 (not at all) to 10 (a lot), and the other evaluates how the module has helped the caregiver to improve several aspects, such as knowing and understanding the problem, understanding emotions, mindfulness of the relationship with their relative, acceptance, family atmosphere, and problem solving, and it is rated from 1 (not at all) to 4 (a lot). Additionally, there is an expectation question only at the end of the first module, where the participants answer the question: “In general, what expectations do you have about the program?”

### Patient measures

#### Sociodemographic interview

***Family Assessment Device – Global Functioning Scale (FAD-GFS)*** [43].

***Depression, Anxiety and Stress Scale (DASS-21)*** [45].

#### Difficulties in emotion regulation scale – Spanish version (DERS) [51]

The authors adapted the scale to spanish and they reduced the items from 36 to 28 with five subscales: emotional lack of control, life interference, lack of emotional attention, emotional confusion, and emotional rejection. All the items have a Likert type design, with a score between 1 and 5, where 1 means “Hardly Ever” and 4 “Usually”, where higher score means more difficulties. Internal consistency was excellent (α = .93) and good test-retest reliability (pl = .74, *p* < .001).

#### Lum emotional availability of parents (LEAP) [52]

It consists of 15 items that measure mothers’ and fathers’ emotional availability perceived by the patient. Items are rated on a 6-point Likert scale ranging from 1 (never) to 6 (always). Internal consistency was excellent in a non-clinical sample for the mother form (α = .96) and the father form (α = .97); and in a clinical sample, for the mother form (α = .92) and the father form (α = .93). This instrument has adequate test–retest reliability for the mother form (*r* = .92) and the father form (*r* = .85). This scale is not validated in Spanish and it will be an objective of this work.

#### Validating and invalidating responses scale (VIRS) [53]

The Validating and Invalidating Responses Scale is a 16-item self-report that evaluates levels of validation and invalidation of caregivers’ responses. This instrument has two subscales: validation and invalidation responses. These two subscales are moderately correlated. Items are rated on a 5-point Likert scale, ranging from 0 (never) to 4 (almost all the time), and higher scores indicate more perceived validation or invalidation from the caregiver who is assessed. There are no psychometric properties available on the VIRS yet. This scale is not validated in Spanish and it will be an objective of this work.

Study measures and evaluation times are summarized in Table 1.

### Data analyses

In order to analyze whether there are differences between the experimental conditions before the application of the treatment, Student’s t tests will be performed for the continuous variables, and chi-square tests for the categorical variables. To compare the effectiveness of the two treatment conditions, we will perform a multivariate analysis of variance for repeated measures (MANOVA) for the variables with subscales, and ANOVA for the single variables, taking the pretreatment, posttreatment and follow-up moments as within-subject factor and the treatment condition (FC vs TAU) as between-subject factor.

Moreover, between-group changes will be computed by calculating standardized effect sizes (Cohen’s d). Finally, we will perform zero-order correlations and linear regression analyses between the measures of the caregivers and the measures of the patients.

Because the trial is still going on, the state of the art in analytic methodology for RCT will be reviewed before analyzing the data, and so variations in the selection of the most appropriate analytic procedures may occur.

## Discussion

FC is an intervention program for relatives of people with BPD that has been adapted to Spanish by our research team. FC was designed to train relatives of people with BPD to improve global family functioning, empowerment, resilience, validation, and mindfulness skills, and decrease grief, burden, hopelessness, and psychological symptoms [25].

The first aim of this study is to provide data from an RCT about the efficacy of this intervention protocol in a Spanish sample of participants consisting of family members of patients with BPD who are treated at clinical centers specializing in the treatment of this disorder, compared to an active condition (TAU). A second objective is to study the acceptability (expectations and opinions) of this program among the participants. Another aim is to analyze whether there are changes in relatives’ burden and psychological symptoms related to the improvement observed in patients. The fourth aim consists of studying whether these changes are related to the family climate. Finally, we will examine the opinions and perceptions of relatives and patients about both intervention protocols.

The study aims to contribute to the existing literature on the efficacy and effectiveness of intervention programs for relatives of BPD patients, specifically FC. In addition, it aims to assess whether the improvements obtained in the relatives are related to those obtained by the patients themselves. Moreover, this study will help to facilitate access to this type of intervention for Spanish-speaking people, which is important due to the lack of options for many people who suffer from this problem, not only in Spain, but also in many countries in South America, in the United States, or in other countries with a significant number of Spanish-speaking citizens. The study will offer data that can be compared to those obtained in other studies carried out in English-speaking countries.

The data obtained in this study can be compared to results obtained in studies with DBT skills protocol programs for relatives. Several studies have found improvements in mental health patients’ relatives and the relationship with their loved ones, but further research is needed. One of the aims of this study is to examine the effect of the treatment components on increasing global family functioning and decreasing burden and distress, which will mean an important change in the research and treatment of relatives of people with BPD. To our knowledge, this is the first RCT study to compare FC to an active condition (TAU) and include a 6-month follow-up.

An important aim of the study is to identify methods to improve access to FC, as well as providing psychological support to everyone who needs it. We are living in a new era in the field of personality disorders, where BPD is given more and more attention. Researchers and clinicians are already crossing the barriers of traditional classifications and treatments, and we can now use these new protocols with significant and encouraging results. The use of the treatment in group format (a more cost-effective format than individual therapy) can help to disseminate and increase the access to these family interventions.

To conclude, in this study, the effectiveness of the application of the FC program will be tested by measuring the acceptability of this program and each specific module in relatives of patients with BPD.

An important strength of this study is that it is the first RCT of FC compared to an active intervention, and it is carried out in a routine clinical care context, an ecological setting. If the hypotheses are confirmed, we expect a fast implementation of FC in these centers and other similar settings. It is also the first study carried out in a Spanish-speaking population, thus facilitating the dissemination of the program in other Spanish-speaking countries or populations.

However, this study has some limitations. We do not expect to have recruitment difficulties because our research team collaborates with different clinical centers, but even so we would have liked to increase the number of centers participating in the study. This was not possible for funding and logistic reasons. Another limitation is that we included a follow-up at 6 months. We would have liked to carry out a long-term follow-up, but due to the conditions of the centers, it is difficult to contact relatives or patients who leave treatment or are discharged.

Finally, the aim of this study is to contribute to the literature on the efficacy of the FC program for relatives of people with BPD. We hope that this study contributes to the exploration of the efficacy and acceptability of programs designed to improve global family functioning and reduce family members’ burden. It will also contribute to improving our understanding of the relationships that may exist between the clinical evolution of the family members receiving the program and the evolution of the patients. If significant results are achieved, there will be an effect on the design and application of future family intervention programs, as a way to improve the overall functioning of the family climate and reduce the burden and distress they face. Finally, this study will allow the possible application of the program to Spanish-speaking populations in other countries.

## Data Availability

It is not possible to share the data because the study is in progress. We are now at the stage of data recruitment.

## Abbreviations

BPD: Borderline personality disorder
FC: Family connections
TAU: Treatment as usual
DBT: Dialectical behavior therapy
RCT: Randomized controlled trial
BAS: Burden assessment scale
FAD-GFS: Family assessment device-global functioning scale
DASS-21: Depression, anxiety and stress scale
FES: Family empowerment scale
QLI-Sp: Quality of life index-spanish version
OTSM: Opinion of treatment scales by modules
DERS: Difficulties in emotion regulation scale
LEAP: Lum emotional availability of parents
VIRS: Validating and invalidating responses scale
S-D: Socio-demographic
BL: Baseline
Post-T: Post-treatment
FU: Follow-up
MANOVA: Multivariate analysis of variance
ANOVA: Analysis of variance
